# Targeting hyaluronan synthesis enhances the therapeutic effectiveness of biologics in inflammatory bowel disease

**DOI:** 10.1172/jci.insight.180425

**Published:** 2025-01-09

**Authors:** Peng Xiao, Zhehang Chen, Xuechun Cai, Wenhao Xia, Xia Liu, Zhangfa Song, Huijuan Wang, Yuening Zhao, Youling Huang, Yu Zhang, Ke Guo, Haotian Chen, Rongbei Liu, Changcheng Meng, Yanfei Fang, Yunkun Lu, Qian Cao

**Affiliations:** 1 Department of Gastroenterology and; 2 Inflammatory Bowel Disease Center, Sir Run Run Shaw Hospital, and; 3 Institute of Immunology, Zhejiang University School of Medicine, Hangzhou, China.; 4 The Key Laboratory for Immunity and Inflammatory Diseases of Zhejiang Province, Hangzhou, China.; 5 ZJU-Hangzhou Global Scientific and Technological Innovation Center, Zhejiang University, Hangzhou, China.; 6 Department of Colorectal Surgery and; 7 Department of General Surgery, Sir Run Run Shaw Hospital, Zhejiang University School of Medicine, Hangzhou, China.

**Keywords:** Gastroenterology, Immunology, Extracellular matrix, Immunotherapy, Inflammatory bowel disease

## Abstract

Although biologics have been revolutionizing the treatment of inflammatory bowel diseases (IBD) over the past decade, a significant number of patients still fail to benefit from these drugs. Overcoming the nonresponse to biologics is one of the top challenges in IBD treatment. In this study, we revealed that hyaluronan (HA), an extracellular matrix (ECM) component in the gut, is associated with nonresponsiveness to infliximab and vedolizumab therapy in patients with IBD. In murine colitis models, inhibition of HA synthase 2–mediated (HAS2-mediated) HA synthesis sensitized the therapeutic response to infliximab. Mechanistically, HA induced the expression of MMP3 in colonic fibroblasts by activating STAT3 signaling, thereby mediating the proteolytic cleavage of multiple IgG1 biologics. Finally, we found that macrophage-derived factors upregulated HAS2 expression in fibroblasts, thereby contributing to infliximab nonresponse. In summary, we identified a pathogenic connection between abnormal ECM remodeling and biologics nonresponse and provided insights for the precise therapy for IBD.

## Abstract 2

This study suggests that inhibiting hyaluronan synthesis is a potential strategy to increase the efficacy of biologics-based therapy in patients with inflammatory bowel disease.

## Graphical abstract

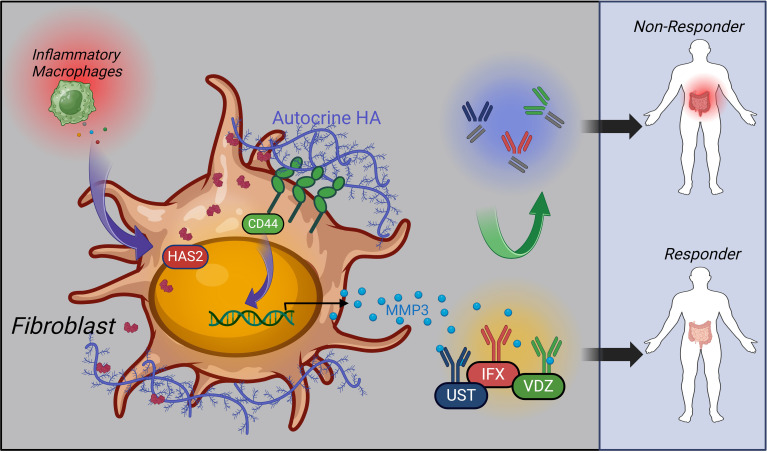

## Introduction

Inflammatory bowel disease (IBD) is a chronic and recurrent gut inflammation with a multifactorial etiology. With the increasing incidence of IBD worldwide, developing effective therapeutic strategies against IBD is becoming an urgent medical demand. The traditional anti-IBD drugs are primarily nonbiological immunosuppressive agents, such as 5-aminosalicylic acid, azathioprine, methotrexate, and corticosteroids. In the past decade, biologics targeting specific inflammatory pathways have dominated the treatment of IBD (1, 2). Biologics refer to drugs produced by living organisms. The most widely used biologics are monoclonal antibodies (mAbs) against inflammatory mediators, including anti–TNF-α mAb (infliximab [IFX]), anti–IL-12/IL-23 mAb (ustekinumab [UST]), and anti-α4β7 integrin mAb (vedolizumab [VDZ]). Compared with traditional drugs, biologics have advantages in their high specificity, high efficacy, and low numbers of adverse effects. However, although the majority of patients with IBD can benefit from biologics, approximately 30% of patients do not generate a therapeutic response after receiving initial treatment, and this is called primary nonresponse (PNR) (3). PNR patients may miss out of the opportunity for timely treatment with appropriate drugs and thus lose the opportunity to recover from IBD. Some patients showed initial clinical improvement but lost the response during the treatment, and they are referred to as having secondary nonresponse (SNR) (4). In addition, their relatively high price makes how to sensitize the effect of biologics when used at a low dosage an attractive question. Disappointingly, current understanding of the mechanisms that determine the effectiveness of biologics is rather limited and thus substantially hinders the prediction or improvement of their therapeutic outcomes.

Herein, we found that, as a ubiquitously distributed extracellular matrix (ECM) constituent, HA deposition in the intestine is associated with an inferior prognosis in patients with IBD treated with IgG1 biologics. Inhibition of HA synthesis by a clinically approved drug, 4-methylumbelliferone (4MU), sensitized the therapeutic effectiveness of IFX in murine colitis models. The underlying mechanisms of HA-induced biologics nonresponse were also explored in this study.

## Results

### Excessive HA synthesis is a hallmark of biologics nonresponse in patients with IBD.

**Figure 1:**
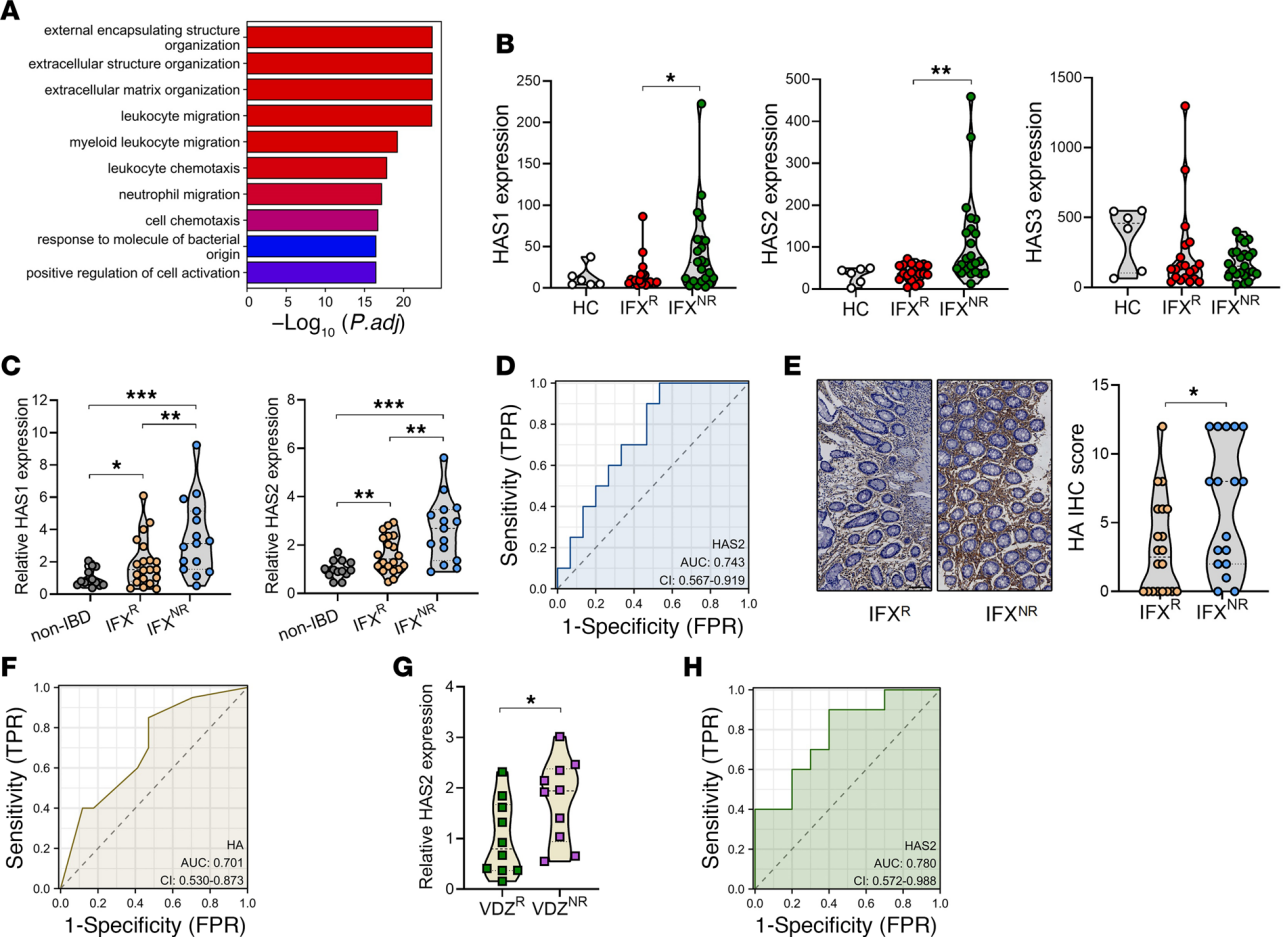
HA deposition is correlated with therapeutic nonresponse to IFX and VDZ in IBD. (**A**) The differentially expressed genes between IFX^R^ (*n* = 20) and IFX^NR^ patients with IBD (*n* = 23) were subjected to GO analysis using the GSE16879 dataset. The samples harvested before IFX treatment were included for analysis. (**B**) The expression of HAS1, -2, and -3 in the intestinal mucosa of IFX^R^ and IFX^NR^ patients with IBD was analyzed using GSE16879 dataset. (**C**) Intestinal mucosa from patients with IBD was collected prior to IFX treatment. The expression of HAS1 and HAS2 was evaluated by QPCR (IFX^R^, *n* = 20; IFX^NR^, *n* = 15). (**D**) Receiver operating characteristic curve (ROC) curve analysis indicated the role of mucosal HAS2 expression in predicting IFX responsiveness. TPR, true positive rate; FPR, false positive rate. (**E**) HA contents were analyzed in IFX^R^ and IFX^NR^ patients with IBD by immunohistochemistry. Original magnification, ×20. (**F**) ROC curve analysis indicating the role of mucosal HA contents in predicting IFX responsiveness. (**G**) Intestinal mucosa from patients with IBD was collected prior to VDZ treatment. HAS2 expression was evaluated by QPCR (VDZ^R^, *n* = 10; VDZ^NR^, *n* = 10). (**H**) ROC curve analysis indicated the role of mucosal HAS2 expression in predicting VDZ responsiveness. *P < 0.05; **P < 0.01; ***P < 0.001. Unpaired, 2-tailed Student’s *t* test was used for **B**, **C**, **E**, and **G**.

In order to dissect the microenvironmental features associated with IFX nonresponsiveness, we first explored a Gene Expression Omnibus (GEO) dataset that profiled gene expression of intestinal mucosa from IFX-responding (IFX^R^) and IFX-nonresponding (IFX^NR^) patients with IBD (5). The Gene Ontology (GO) enrichment analysis revealed that the differentially expressed genes were highly enriched in the pathways related to ECM remodeling. One of the top pathways identified was “extracellular structure organization” (Figure 1A). In various mammalian tissues including colons, hyaluronan (HA) is the major ECM component. We found that, among the 3 HA synthases (HASs) that catalyze HA synthesis, the expression of HAS1 and HAS2 was significantly upregulated in IFX^NR^ patients compared with that in IFX^R^ patients, and HAS3 expression was comparable between the two groups (Figure 1B). On the other hand, the levels of genes encoding hyaluronidases that catalyze the degradation of HA were either unchanged or decreased in IFX^NR^ patients compared with IFX^R^ patients (Supplemental Figure 1; supplemental material available online with this article; https://doi.org/10.1172/jci.insight.180425DS1).

To validate these results, we collected intestinal mucosa from patients with IBD (including patients with ulcerative colitis and Crohn’s disease) before IFX treatment and divided samples into IFX^R^ and IFX^NR^ groups according to therapeutic outcomes. Compared with that in the IFX^R^ group, the expression of both HAS1 and HAS2 was significantly higher in IFX^NR^ group (Figure 1C). Under physiologic conditions, HA exists as a high-molecular-weight form, which is predominantly synthesized by HAS2 (6–10). We observed that mucosal HAS2 expression showed high accuracy in distinguishing IFX^R^ and IFX^NR^ patients, with an area under the receiver operating characteristic curve (AUC) of 0.743 (Figure 1D). Additionally, immunohistochemistry staining also revealed significantly higher intestinal HA production in IFX^NR^ patients (Figure 1E). The AUC of HA scores was 0.701 (Figure 1F). The levels of TNF-α in the colonic mucosa were not significantly different between the IFX^R^ and IFX^NR^ groups (Supplemental Figure 2).

We further assessed HAS2 expression in VDZ-responding (VDZ^R^) or -nonresponding (VDZ^NR^) IBD mucosa collected before treatment. Similar to that in IFX cohort, HAS2 expression was significantly higher in VDZ^NR^ patients than in VDZ^R^ patients (Figure 1G). The AUC of HAS2 expression was 0.780 (Figure 1H), indicating a good diagnostic performance. Therefore, we identified HA oversynthesis as a potentially novel hallmark of IFX and VDZ nonresponsiveness in patients with IBD.

### Inhibition of HA synthesis overcomes IFX nonresponse in colitis.

**Figure 2:**
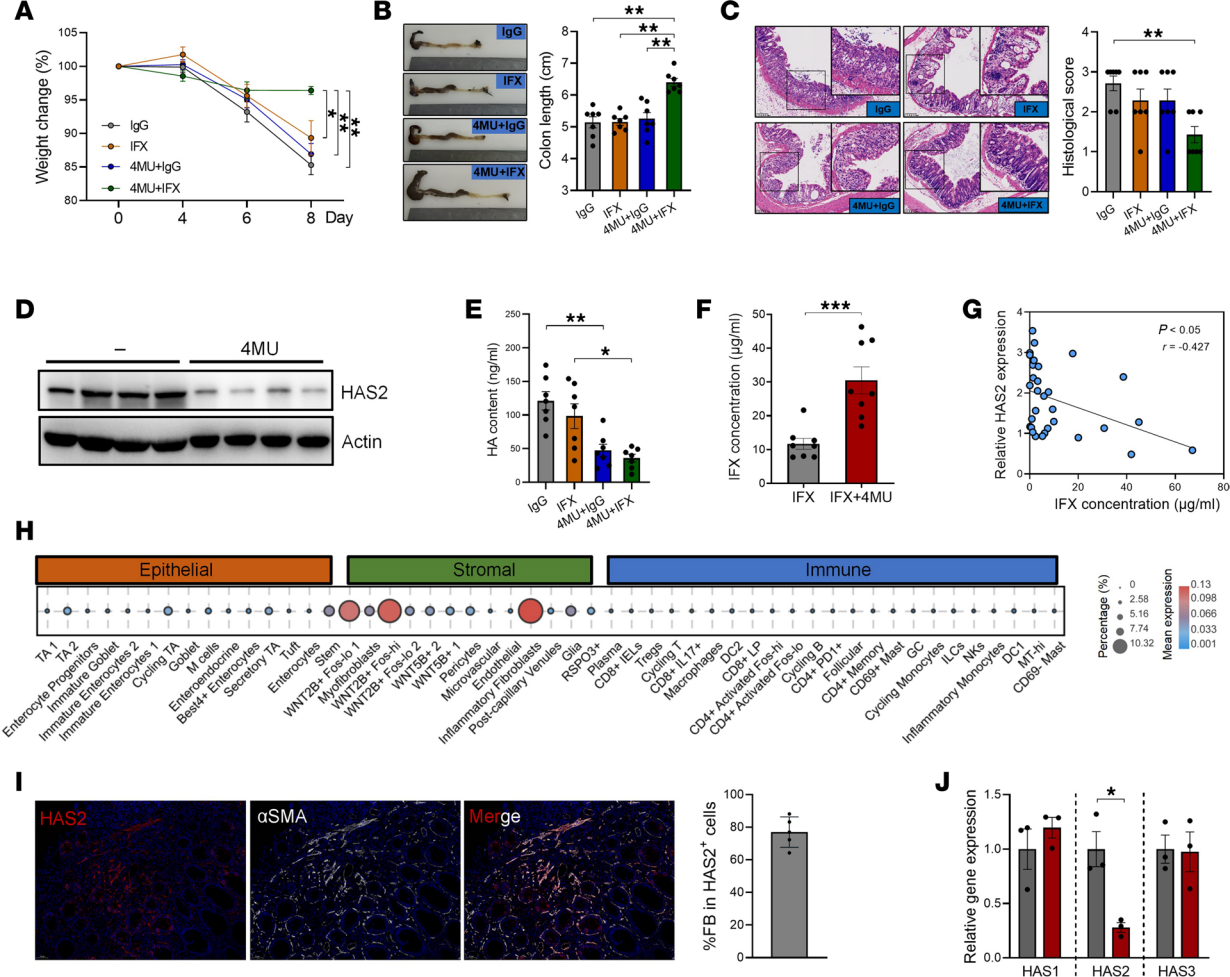
HAS2 inhibition sensitized IFX treatment in colitis. (**A**) Mice given 2.5% DSS were treated with IFX, 4MU, or a combination (*n* = 7/group). Body weight changes were monitored. (**B**) Colon length was measured on day 8. (**C**) Histological damage was evaluated by H&E staining. Original magnification, ×15; ×27 (insets). (**D**) Protein levels of HAS2 in colon tissues were evaluated by immunoblotting. (**E**) HA contents in the colons of colitic mice were evaluated by ELISA. (**F**) The concentrations of serum IFX in colitic mice were evaluated by ELISA. (**G**) The correlation between pretreatment mucosal HAS2 expression and posttreatment serum IFX concentrations in patients with IBD was analyzed. (**H**) The cell-specific expression of HAS2 was analyzed in human colons using the Single Cell Portal tool (accession SCP259; https://singlecell.broadinstitute.org/single_cell/study/SCP259/intra-and-inter-cellular-rewiring-of-the-human-colon-during-ulcerative-colitis). (**I**) The colocalization of HAS2 and αSMA^+^ fibroblasts was evaluated and quantified in IBD mucosa by immunofluorescence staining. Original magnification, ×20. (**J**) HcFBs were treated with 2 mM 4MU for 24 hours. The levels of HAS1, -2, and -3 were evaluated by QPCR. *P < 0.05; **P < 0.01; ***P < 0.001. Unpaired, 2-tailed Student’s *t* test was used for **F** and **J**; ANOVA followed by Kruskal-Wallis test and Dunn’s correction was used for **A**–**C** and **E**; and Spearman’s rank correlation test was used for **G**.

The aforementioned clinical evidence prompts us to investigate the effect of HA production on the therapeutic effectiveness of IFX. For this purpose, we adopted a murine DSS-induced colitis model and treated mice with IFX or 4MU (a HAS2 inhibitor) alone or in combination. As shown in Figure 2A, monotherapy with IFX or 4MU marginally improved the weight loss of colitic mice. However, the combined treatment of IFX plus 4MU conferred robust protection against colitis-induced weight loss and colon shortening (Figure 2, A and B). In addition, IFX significantly alleviated histological damage in the presence of 4MU but not when used alone (Figure 2C). The levels of proinflammatory cytokines, such as IL-6, IL-1β, CXCL10, and IFN-γ, were also significantly lower in the colon tissues from combination treatment group than the other groups (Supplemental Figure 3). These results confirmed that inhibiting HA synthesis sensitized the efficacy of IFX therapy in colitis. Encouragingly, 4MU is an oral drug that has already been approved in Europe and Asia (11, 12), highlighting its potential for clinical translatability.

As expected, 4MU treatment remarkably reduced HAS2 expression and HA production in the inflamed colons (Figure 2, D and E, and Supplemental Figure 4). Intriguingly, we noticed that the serum concentrations of IFX were significantly higher in 4MU-treated mice than in control mice (Figure 2F). Moreover, the mucosal expression of HAS2 before treatment was inversely correlated with posttreatment serum IFX concentrations from the matched patients with IBD (Figure 2G), suggesting that HA deposition might impair the stability of IFX.

To explore the cell-specific expression of HAS2, we examined a single-cell RNA sequencing (scRNA-Seq) dataset (Single Cell Portal tool, https://singlecell.broadinstitute.org/single_cell, accession SCP259) and found that fibroblasts expressed the highest level of HAS2 in the human colon (Figure 2H). Immunofluorescence staining further confirmed that HAS2 exhibited substantial colocalization with αSMA^+^ fibroblasts in IBD mucosa (Figure 2I). We then isolated primary human colon fibroblasts (hcFBs) and treated them with 4MU. As shown in Figure 2J, 4MU significantly downregulated the expression of HAS2 but not HAS1 and HAS3 in hcFBs. Therefore, interfering with HAS2-mediated HA synthesis in fibroblasts sensitizes IFX therapy in colitis.

### HA drives the production of MMP3 in fibroblasts through activating STAT3 signaling.

**Figure 3:**
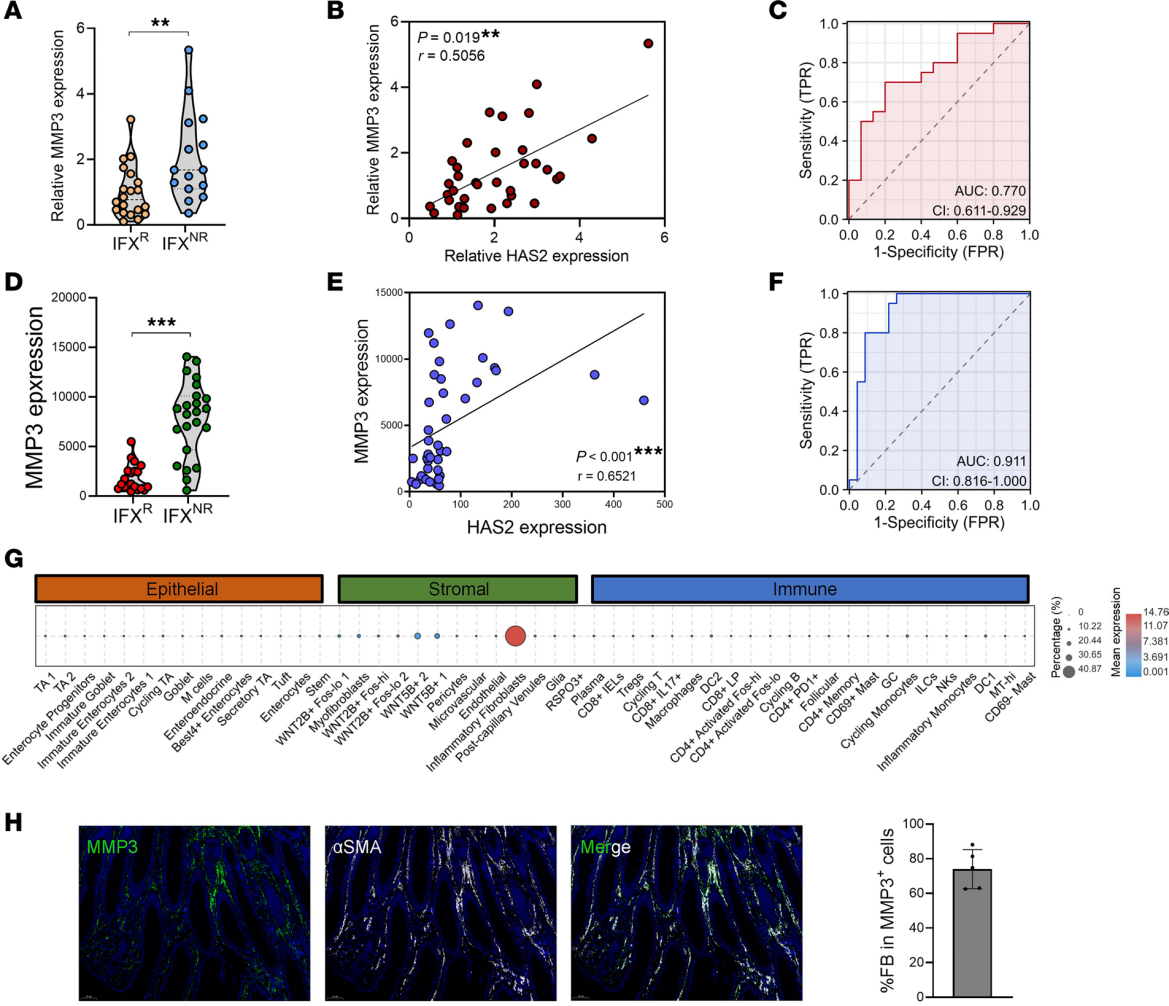
The correlation between mucosal HAS2 and MMP3 expression in patients with IBD. (**A**–**C**) Intestinal mucosa from patients with IBD was harvested before IFX treatment (IFX^R^, *n* = 20; IFX^NR^, *n* = 15). The expression of MMP3 was evaluated by QPCR (**A**), the correlation between mucosal HAS2 and MMP3 expression was analyzed (**B**), and ROC curve analysis showed the role of MMP3 expression in predicting IFX responsiveness (**C**). (**D**–**F**) Mucosal MMP3 expression (**D**), its correlation with HAS2 expression (**E**), and ROC curve of MMP3 (**F**) were validated using the GSE16879 dataset (IFX^R^, *n* = 20; IFX^NR^, *n* = 23). (**G**) Cell-specific expression of MMP3 was analyzed in human colons using Single Cell Portal tool (accession SCP259; https://singlecell.broadinstitute.org/single_cell/study/SCP259/intra-and-inter-cellular-rewiring-of-the-human-colon-during-ulcerative-colitis). (**H**) The colocalization of MMP3 and αSMA^+^ fibroblasts was evaluated and quantified in IBD mucosa by immunofluorescence staining. Original magnification, ×20. **P < 0.01; ***P < 0.001. Unpaired, 2-tailed Student’s *t* test was used for **A** and **D**, and Spearman’s rank correlation test was used for **B** and **E**.

As a humanized IgG1 antibody, IFX can be proteolytically cleaved by MMP3 (13). We found that IFX^NR^ patients had significantly higher MMP3 levels than IFX^R^ patients (Figure 3A). In addition, there was a significant positive correlation between mucosal MMP3 and HAS2 expression in patients with IBD (Figure 3B). The accuracy of mucosal MMP3 expression in distinguishing IFX^R^ and IFX^NR^ patients was evidenced by an AUC of 0.770 (Figure 3C). Through analyzing a GEO dataset (https://www.ncbi.nlm.nih.gov/geo, accession GSE16879), the higher MMP3 expression in IFX^NR^ patients, the positive correlation between mucosal HAS2 and MMP3 levels, and the excellent diagnostic value of MMP3 expression (AUC = 0.911) were further validated (Figure 3, D–F).

**Figure 4:**
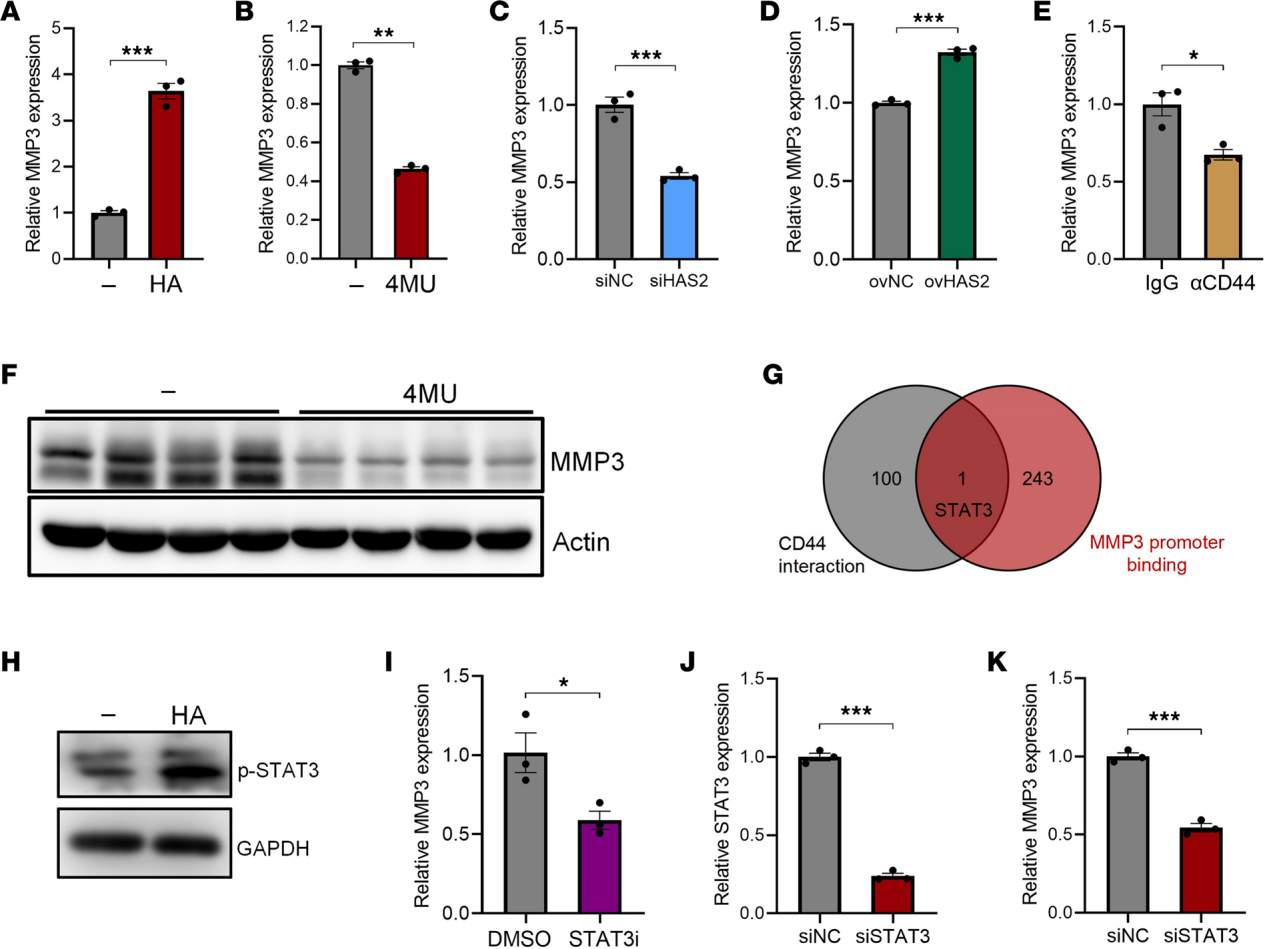
HA upregulates MMP3 expression in colonic fibroblasts in a STAT3-dependent manner. (**A**) HcFBs were treated with 100 μg/mL HA for 24 hours. MMP3 expression was evaluated by QPCR. (**B**) HcFBs were treated with 2 mM 4MU for 24 hours. MMP3 expression was evaluated by QPCR. (**C**) HcFBs were transfected with siHAS2 for 72 hours. MMP3 expression was evaluated by QPCR. NC, negative control. (**D**) HcFBs were transfected with HAS2 overexpression vectors for 72 hours. MMP3 expression was evaluated by QPCR. (**E**) HcFBs were treated with 1 μg/mL anti-CD44 antibody for 24 hours. MMP3 expression was evaluated by QPCR. (**F**) Protein levels of MMP3 in the colon tissues of colitic mice were evaluated by immunoblotting. (**G**) Venn diagram showing proteins that could potentially interact with both human MMP3 promoter and CD44. (**H**) HcFBs were treated with 100 μg/mL HA for 24 hours. STAT3 phosphorylation was evaluated by immunoblotting. (**I**) HcFBs were treated with 5 μM STAT3-IN-1 (a STAT3 inhibitor) for 24 hours. MMP3 expression was evaluated by QPCR. (**J**) STAT3 expression was silenced in hcFBs through siSTAT3 transfection. The levels of STAT3 were evaluated by QPCR. (**K**) The expression of MMP3 was evaluated in control- or STAT3-silenced hcFBs. *P < 0.05; **P < 0.01; ***P < 0.001. Unpaired, 2-tailed Student’s *t* test was used for **A**–**E** and **I**–**K**.

Next, we performed scRNA-Seq analysis to identify MMP3-producing cells in the colon. Similar to HAS2, MMP3 expression was also specifically found in fibroblasts (Figure 3G). Immunofluorescence staining confirmed that αSMA^+^ fibroblasts were the primary MMP3-producing cells in IBD mucosa (Figure 3H), suggesting that fibroblast-derived HA might stimulate MMP3 production in an autocrine manner. To test this hypothesis, we treated hcFBs with HA and found that HA significantly increased the expression of MMP3 in fibroblasts (Figure 4A). In contrast, HAS2 inhibition by 4MU significantly decreased MMP3 expression in hcFBs (Figure 4B). In agreement, HAS2 silence significantly reduced the expression of MMP3 in hcFBs (Figure 4C), whereas HAS2 overexpression caused the opposite effect (Figure 4D). The natural receptor of HA is CD44 (14). We treated fibroblasts with a blocking antibody against CD44 that prevents its interaction with HA (15). As expected, anti-CD44 treatment significantly reduced MMP3 levels in fibroblasts (Figure 4E). Consistent with the decreased HA deposition in 4MU-treated mice, the levels of MMP3 in colon tissues were substantially reduced by 4MU (Figure 4F).

To further elucidate the underlying mechanism behind HA/CD44-induced MMP3 upregulation, we identified 244 proteins that were predicted to bind to *MMP3* promoter in the UCSC/JASPAR database (https://genome.ucsc.edu/cgi-bin/hgTrackUi?db=hg19&g=jaspar). On the other hand, 101 proteins were found to interact with CD44 using the STRING database (https://string-db.org/). Notably, the transcription factor STAT3 was the sole overlapping protein in both datasets (Figure 4G). Indeed, HA stimulation enhanced STAT3 phosphorylation in hcFBs (Figure 4H). When hcFBs were treated with a STAT3 inhibitor, the expression of MMP3 was significantly reduced (Figure 4I). We further silenced STAT3 expression in hcFBs (Figure 4J) and found that MMP3 expression was significantly reduced upon STAT3 silencing (Figure 4K), indicating that STAT3 is involved in the HA-induced transcriptional activation of MMP3.

### MMP3 inhibition improves the therapeutic effectiveness of IFX in colitis.

**Figure 5:**
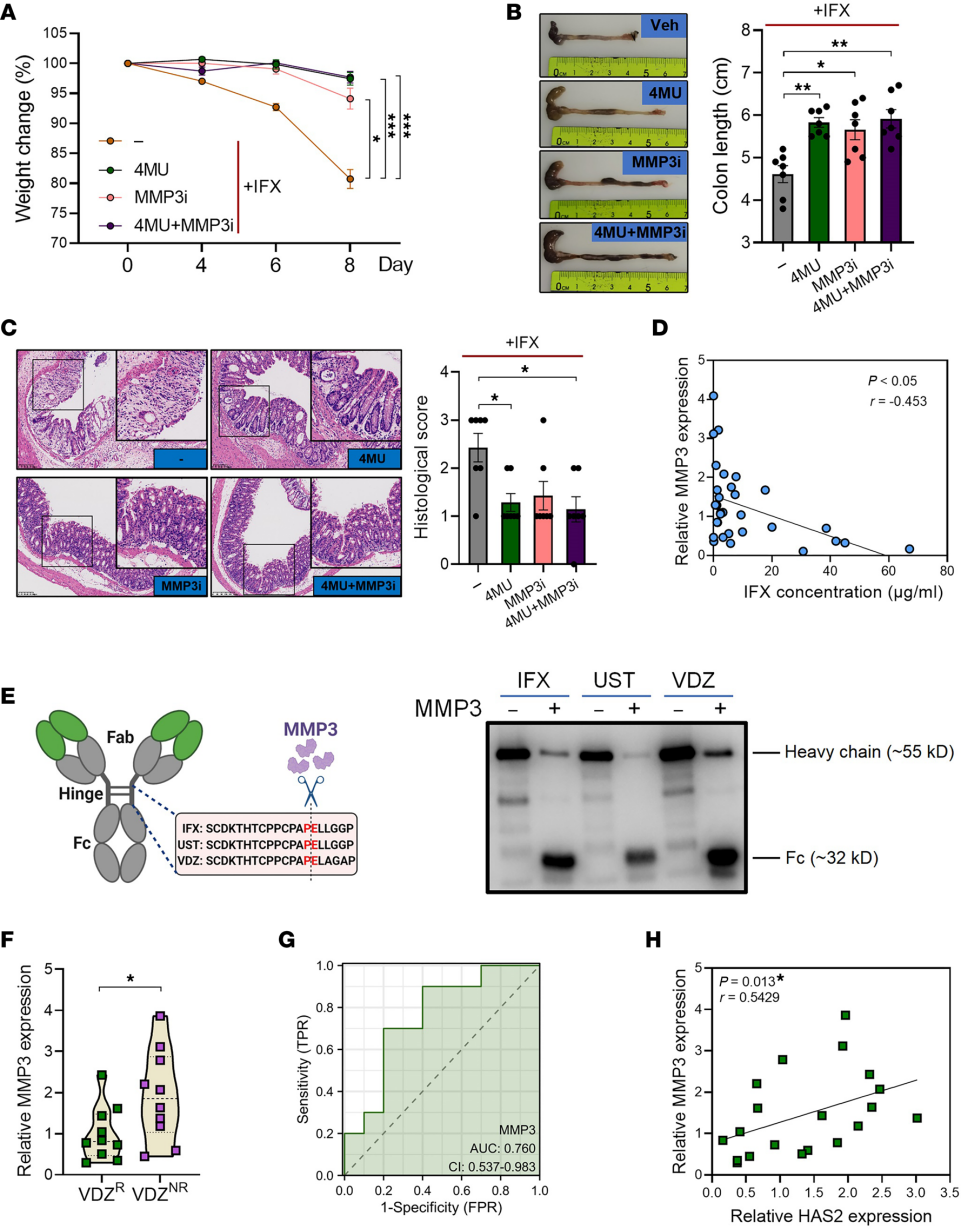
MMP3 contributes to IFX nonresponse by mediating its cleavage. (**A**) Mice given 2.5% DSS were treated with 2 mg/mL 4MU (in drinking water), 160 μg NNGH (i.p. injection, every other day), or a combination (*n* = 7/group). Body weight changes were monitored. (**B**) Colon length was measured on day 8. (**C**) Histological damage was evaluated by H&E staining. Original magnification, ×15; ×27 (insets). (**D**) The correlation between pretreatment mucosal MMP3 expression and posttreatment serum IFX concentration in patients with IBD was analyzed. (**E**) The structure of human IgG1 antibody and the cleavage sites of MMP3 in IFX, UST, and VDZ (left). IFX, UST, and VDZ were incubated with 10 μg/mL recombinant MMP3 for 24 hours, and the cleavage of antibodies was confirmed by immunoblotting (right). (**F**–**H**) Intestinal mucosa from patients with IBD was collected prior to VDZ treatment (VDZ^R^, *n* = 10; VDZ^NR^, *n* = 10), and MMP3 expression was evaluated by QPCR (**F**), ROC curve analysis showed the role of MMP3 expression in predicting VDZ responsiveness (**G**), and the correlation between mucosal levels of HAS2 and MMP3 was analyzed (**H**). *P < 0.05; **P < 0.01; ***P < 0.001. Unpaired, 2-tailed Student’s *t* test was used for **F**; ANOVA followed by Kruskal-Wallis test and Dunn’s correction was used for **A**–**C**; Spearman’s rank correlation test was used for **D** and **H**.

To determine the effect of MMP3 on the effectiveness of IFX, we treated colitic mice with 4MU, MMP3 inhibitor (NNGH), or a combination. Both 4MU and NNGH significantly sensitized the therapeutic efficacy of IFX, with no obvious apparent effect when used in combination (Figure 5, A–C). Similar to that of HAS2, the mucosal expression of MMP3 before treatment was significantly negatively correlated with posttreatment serum IFX concentrations in patients with IBD (Figure 5D). Immunoblotting results confirmed the proteolytic cleavage of IFX by recombinant MMP3 (Figure 5E). Surprisingly, we did not observe a reduction in IFX concentration in the culture supernatant of hcFB after MMP3 cleavage (data not shown). This might be because the cleaved IFX fragments can still be recognized in the in vitro system, whereas they have reduced in vivo stability compared with complete IFX, because smaller antibody fragments lacking the Fc domain generally have a shorter circulating half-life than intact antibodies (16, 17).

Apart from IFX, we found that the MMP3 cleavage site also exists in the hinge region of UST and VDZ, which are biologics. Indeed, UST and VDZ were proteolytically cleaved after incubation with MMP3 (Figure 5E). In line with this result, VDZ^NR^ patients with IBD had significantly higher MMP3 expression than VDZ^R^ patients (Figure 5F). The AUC of MMP3 expression was 0.760 (Figure 5G), indicating a good diagnostic performance. Similar to that in the IFX cohort, we observed a significantly positive correlation between mucosal expression of HAS2 and MMP3 in VDZ-treated patients (Figure 5H). The effect of HA on the effectiveness of VDZ or UST was not evaluated in murine colitis models because these mAbs cannot bind to murine α4β7 or IL-12.

In summary, HA upregulates MMP3 production by fibroblasts, which leads to a lack of response to IgG1 biologics in IBD.

### Macrophages drive fibroblast HAS2 expression and correlate with nonresponse to biologics.

**Figure 6:**
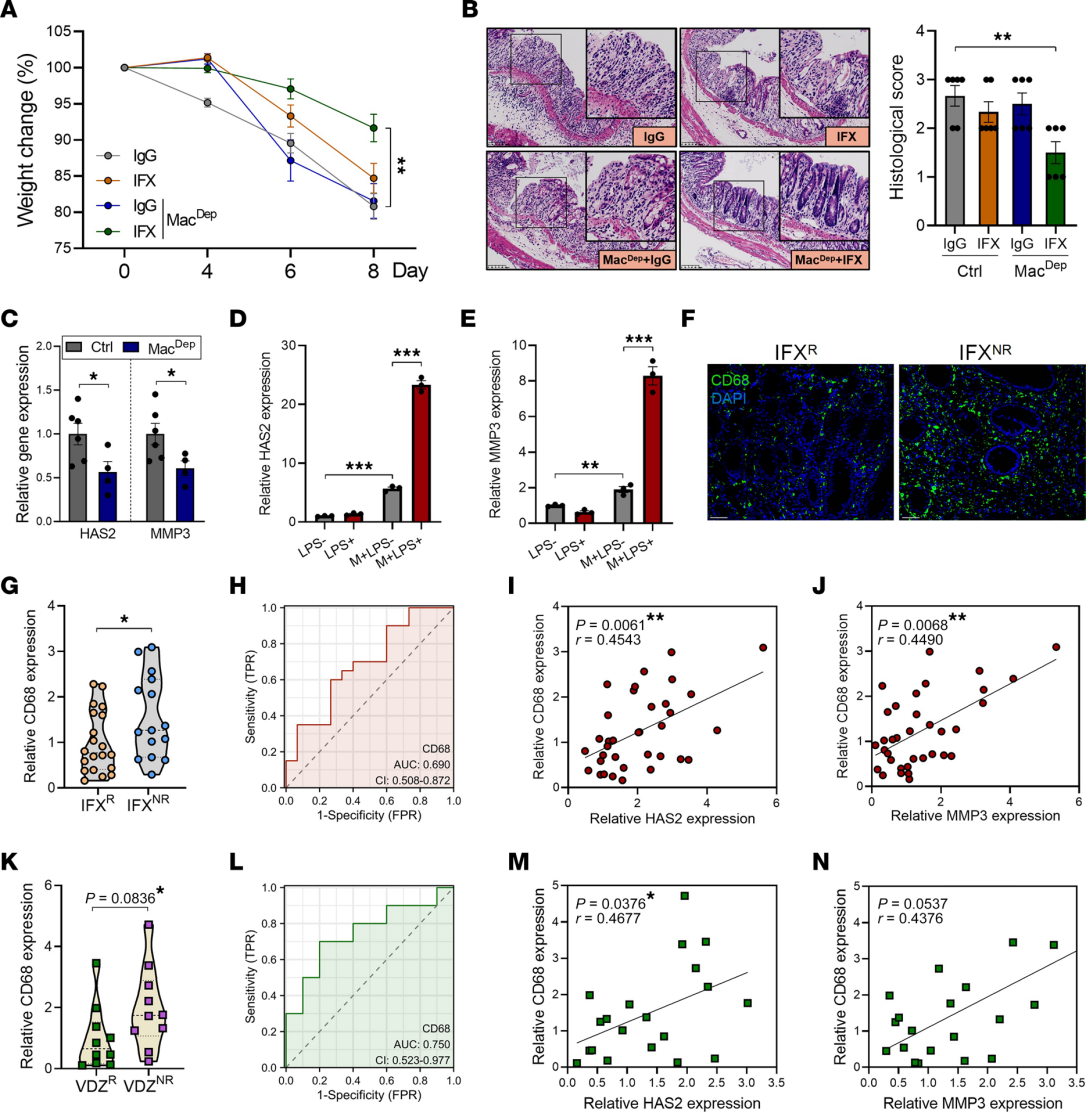
Macrophages upregulate HAS2 expression in fibroblasts. (**A** and **B**) Mice were given 2.5% DSS after macrophage depletion with clodronate liposomes (*n* = 6/group), and body weight changes were monitored (**A**) and histological damage was evaluated by H&E staining (**B**). Original magnification, ×15; ×27 (insets). (**C**) The expression of HAS2 and MMP3 in colon tissues was evaluated by QPCR on day 8 (*n* = 4–6/group). (**D** and **E**) HcFBs were treated with M^SN^ or M+LPS^SN^ for 24 hours, and the expression of HAS2 and MMP3 was evaluated by QPCR. (**F**) The infiltration of CD68^+^ macrophages was evaluated in the colonic mucosa of IFX^R^ and IFX^NR^ patients by immunofluorescence staining. Scale bar: 50 μm. (**G**) The levels of pretreatment mucosal CD68 expression were evaluated in IFX^R^ and IFX^NR^ patients with IBD by QPCR (IFX^R^, *n* = 20; IFX^NR^, *n* = 15). (**H**) ROC curve analysis indicated the role of mucosal CD68 expression in predicting IFX responsiveness. (**I** and **J**) The correlations between mucosal CD68 and HAS2 (**I**) or MMP3 (**J**) expression in IFX cohort were analyzed by Spearman’s rank correlation test. (**K**) The levels of pretreatment mucosal CD68 expression were evaluated in VDZ^R^ and VDZ^NR^ patients with IBD by QPCR (VDZ^R^, *n* = 10; VDZ^NR^, *n* = 10). (**L**) ROC curve analysis indicated the role of mucosal CD68 expression in predicting VDZ responsiveness. (**M** and **N**) The correlations between mucosal CD68 (**M**) and HAS2 (**N**) expression in the VDZ cohort was analyzed by Spearman’s rank correlation test. *P < 0.05; **P < 0.01; ***P < 0.001. Unpaired, 2-tailed Student’s *t* test was used for **C**–**E**, **G**, and **K**; ANOVA followed by Kruskal-Wallis test and Dunn’s correction was used for **A** and **B**; and Spearman’s rank correlation test was used for **I**, **J**, **M**, and **N**.

The function of fibroblasts is profoundly influenced by macrophages (18–20), which represent the most abundant immune cells in the colon (21). This prompts us to question whether macrophages are involved in regulating the HAS2-MMP3 axis in fibroblasts. Therefore, we depleted macrophages in colitic mice using clodronate liposomes. Although macrophages serve as major sources of inflammatory cytokines, the depletion of macrophages did not obviously alter the severity of colitis, presumably due to the crucial role of macrophages in mediating bacterial clearance and mucosal healing in colitis (22, 23). Nevertheless, the therapeutic response to IFX was significantly improved in macrophage-depleted mice (Figure 6, A and B). Importantly, macrophage depletion led to a significant decrease in HAS2 and MMP3 levels in the colon, indicating that macrophages promote HAS2/MMP3 expression during inflammation (Figure 6C). Next, we treated hcFBs with culture supernatants from primary human macrophages (M^SN^). Notably, the levels of HAS2 and MMP3 in hcFBs were significantly increased upon M^SN^ treatment. Intriguingly, when hcFBs were treated with culture supernatants from LPS-stimulated macrophages, the expression of HAS2 and MMP3 was further upregulated. On the other hand, LPS failed to directly increase their expression in hcFBs (Figure 6, D and E). These findings suggest that macrophages, particularly inflammatory macrophages, stimulated the HAS2-MMP3 axis in colonic fibroblasts.

In the IFX cohort, we found higher infiltration of CD68^+^ colonic macrophages in IFX^NR^ patients than in IFX^R^ patients (Figure 6F). In addition, the expression levels of CD68 (a macrophage-specific marker) were significantly increased in IFX^NR^ group (Figure 6G). The AUC of CD68 expression was 0.690 (Figure 6H). In line with the effect of macrophages on HAS2 induction, there was a significant positive correlation between mucosal CD68 and HAS2 expression in IFX cohort (Figure 6I). Not surprisingly, CD68 also significantly correlated with MMP3 expression (Figure 6J). Similarly, in the VDZ cohort, CD68 expression was higher in VDZ^NR^ patients than in VDZ^R^ patients (although no statistical significance was achieved, *P* = 0.0836; Figure 6K), with an AUC of 0.750 (Figure 6L), and it was significantly positively correlated with mucosal HAS2 expression (Figure 6M). Moreover, a positive correlation was observed between the expression of CD68 and MMP3 in the VDZ cohort (Figure 6N).

Collectively, the crosstalk between macrophages and fibroblasts promotes the pathological HAS2-MMP3 cascade, which undermines the therapeutic responses of IgG1 biologics.

## Discussion

PNR can be a source of great frustration for patients taking IBD biologic therapies, the current understanding of the mechanisms behind PNR is quite limited. In contrast with SNR, the production of antidrug antibodies is not considered a primary cause for PNR. Here, we uncovered what we believe to be a novel mechanism that connects dysregulated ECM remodeling with the nonresponsiveness to IgG1 mAbs. Although the present study only investigated the effect of HA on PNR, it can be speculated that HA also contributes to SNR since it directly cleaves IgG1 mAbs. We are currently investigating this hypothesis in ongoing studies.

Under physiological conditions, HA is essential in supporting embryonic development and organ structural stability. However, excessive HA synthesis is observed in many pathological contexts and promotes progression of diseases, such as cancer, inflammation, and autoimmune diseases (24, 25). Encouragingly, targeting HA synthesis can be achieved by the application of 4MU, a clinically approved drug with proven safety in humans (11). Since MMP3 can cleave multiple IgG1 antibodies, including IFX, UST, and VDZ, the HA-induced therapeutic nonresponse appears to be a common mechanism for IgG1 mAb–based IBD treatment. Indeed, in our work, high HAS2 expression was associated with PNR in both IFX and VDZ cohorts. Data about UST are lacking due to sample availability. In addition, we were unable to test the impact of 4MU on the effectiveness of UST and VDZ in animal colitis models as they do not bind to murine IL-12 and α4β7.

In comparison with our results, a previous study reported that 4MU treatment aggravated colitis-induced weight loss, whereas its effects on colon length and the production of inflammatory cytokines were not obvious (26). According to our preliminary experiments, high-dosage 4MU administration led to a reduction in body weigh in the absence of colitis. Although the detailed protocol for 4MU intervention was not provided in the aforementioned study, we speculate that different treatment strategies for 4MU may affect its effect on colitis. On the other hand, exogenous HA was reported to accelerate the recovery of colitis without affecting the acute phase of colitis (27). As HA is a very heterogeneous molecule, the endogenous and exogenously supplemented HA may have differences in terms of tissue distribution, concentration, molecular weight, molecular structure, and biological function. All of these variables could potentially affect the effect of HA on colitis. Therefore, the exact influence of HA and 4MU on the development or treatment of colitis need further comprehensive elucidation. In addition, although 4MU is a clinically approved drug, it also has off-target effects such as interfering with the production of glycosaminoglycans, through acting as a competitive substrate for UDP-glucuronosyltransferases (28). Therefore, the potential adverse effects of 4-MU could be carefully considered.

In our study, we revealed the potential values of mucosal HA content or HAS2/MMP3 expression in predicting the therapeutic outcomes related to use of IgG1 mAbs before treatment, suggesting that patients with IBD with high HA or HAS2/MMP3 expression might be less likely to benefit from treatment. A previous study has investigated the association between serum MMP3 levels and the therapeutic response to IFX, and it found that IFX^NR^ patients had higher serum MMP3 levels than IFX^R^ patients after but not before IFX treatment (29). Here, we showed that the pretreatment mucosal MMP3 expression predicted the responsiveness to IFX and VDZ therapy. Since the severity of intestinal inflammation was different between IFN^R^ and IFX^NR^ groups after treatment, the difference in MMP3 production might be a secondary effect to the reduced inflammation. Additionally, compared with the serum levels, the MMP3 levels in the inflamed colons are possibly more reliable in reflecting the local production of MMP3.

An intriguing discovery in the current study is that macrophage-derived factors stimulated HAS2 expression in fibroblasts. Through a simplified system, macrophages can be categorized into an M1 proinflammatory subset and an M2 antiinflammatory phenotype (30). In the context of intestinal inflammation, lamina propria macrophages are activated by the invading bacteria and then differentiate into a M1-like phenotype with a potent capacity to produce inflammatory cytokines (31). Herein, we showed that LPS-activated macrophages (M1 macrophages) exhibited a higher capacity to stimulate HAS2 expression in fibroblasts, which undermines the treatment of IgG1 mAbs. Whether there exists a dominant cytokine in macrophage supernatant that upregulates HAS2 expression in fibroblasts requires further identification. Emerging evidence has unveiled the dual effect of macrophages on the responsiveness of biologics therapy. For example, IFX^NR^ patients with IBD exhibited increased infiltration of inflammatory macrophages compared with IFX^R^ patients (32). Macrophage-derived TNF-α induced endoplasmic reticulum stress in intestinal epithelial cells, leading to the diminished effectiveness of anti–TNF-α antibody (33). Similarly, VDZ^R^ patients showed a shift from M1 to M2 macrophages in the gut, a phenomenon that was not observed in VDZ^NR^ patients (34). In contrast, macrophage IL-10 signaling was reported to be required for the therapeutic response of IFX (35). In this work, the depletion of macrophages sensitized the efficacy of IFX treatment, suggesting that the overall effect of macrophages on biologics therapy may be detrimental. Interestingly, the macrophage-mediated HAS2 expression in fibroblasts may generate a reciprocal effect, as HA also regulates the inflammatory activities of macrophages (36, 37).

In summary, this study suggests that targeting HA synthesis could potentially improve the therapeutic effectiveness in patients with IBD undergoing IgG1 biologics treatment.

## Methods

### Sex as a biological variable.

Our study examined clinical samples from male and female patients. Sex was not considered as a biological variable. For animal experiments, male mice were used, because, according to previous reports and our own experience, male mice were more susceptible to the DSS-induced colitis model than female mice (38, 39).

### Murine colitis model.

The DSS-induced colitis model was established as we previously described (39). For 4MU treatment, 4MU (M1381, Sigma-Aldrich) was dissolved in drinking water at a final concentration of 2 mg/mL, starting 1 week before DSS challenge. For IFX treatment, mice were given 5 mg/kg IFX (Janssen-Cilag AG) by i.p. injection, starting on day 0 of DSS (160110, MP Biomedicals) challenge, and injections were performed every other day. For MMP3 inhibition, mice were i.p. injected with 160 μg NNGH (CAY16886, Cayman Chemical) starting on day 0 of DSS challenge, and injections were performed every other day.

### IBD samples.

Intestinal mucosa from patients with IBD and individuals without IBD was collected at SRRSH IBD Biobank in China, Sir Run Run Shaw Hospital, Zhejiang University School of Medicine. Samples were harvested before IFX or VDZ treatment. Serum samples of patients with IBD were collected 4 weeks after IFX treatment. The PNR to IFX or VDZ was assessed as previously described (40–43). Basic patient information is listed in Supplemental Table 1. Experiments were performed under the approval of the Medical Ethics Committee of Sir Run Run Shaw Hospital, Zhejiang University School of Medicine (no. 20210210-24). Informed consent was obtained from all participants.

### Isolation of hcFBs.

HcFBs were isolated from the resected, distant noncancerous colon tissues of patients with colorectal cancer. Tissues were rinsed 3 times with PBS containing 100 U/mL penicillin and 100 μg/mL streptomycin (Thermo Fisher Scientific), cut into approximately 1 mm^3^ fragments, and further washed with 10 mL PBS 3 times. After centrifugation at 400 *g* for 5 minutes, colon tissues were resuspended in 1 mL DMEM medium containing 50% FBS (S-FBS-SA-015, Serana Europe) and placed into a 6 cm dish to allow tissues to adhere to the bottom of the well. Seven days later, fresh DMEM medium containing 50% FBS was added onto the tissues. Fibroblasts were cultured until they reached approximately 80% confluence and were used for the following experiments. The protocols were approved by the Medical Ethics Committee of Sir Run Run Shaw Hospital, Zhejiang University School of Medicine (no. 20220209-256). Informed consent was obtained from all participants.

### The treatment of hcFBs.

Isolated hcFBs were stimulated with 100 μg/mL exogenous HA (924474, Sigma-Aldrich) and 2 mM 4MU for 24 hours. For HA blocking, hcFBs were treated with 50 μg/mL anti-CD44 antibody (NBP2-22530, Novus Biologicals) for 24 hours.

### Fibroblast transfection.

HcFBs were seeded into a 24-well plate. When cell confluence reached approximately 70%–80%, cell transfection was performed using Lipofectamine 3000 Transfection Reagent (L3000001, Thermo Fisher Scientific) according to the manufacturer’s protocol. Plasmid vectors containing HAS2 coding sequence, HAS2 siRNA, and STAT3 siRNA were constructed by GenePharma Technology. Cells were harvested for analysis 3 days after transfection.

### Quantitative PCR.

Quantitative PCR (QPCR) was performed as we previously described (39). Primer sequences are listed in Supplemental Table 2.

### Immunoblotting.

Immunoblotting was performed as we previously described (33). The following antibodies were used: anti-HAS2 (sc-514737, Santa Cruz Biotechnology), anti-MMP3 (sc-21732, Santa Cruz Biotechnology), anti-phospho STAT3 (9145, Cell Signaling Technology), anti–β-Actin (4970S, Cell Signaling Technology), and anti-GAPDH (2118S, Cell Signaling Technology). Anti-mouse IgG (A0216, Beyotime) or anti-rabbit IgG (A0208, Beyotime) were used as secondary antibodies.

### Antibody cleavage.

Recombinant human MMP-3 (513-MP, R&D Systems) and recombinant mouse MMP-3 (548-MM, R&D Systems) were activated following the procedures provided by the manufacturer. Activated MMP3 (10 μg/mL) was incubated with IFX (Janssen-Cilag AG), UST (Janssen-Cilag AG), or VDZ (Takeda Pharmaceutical Company Limited) for 24 hours at 37°C in TCNB buffer. The recipe of TCNB buffer was as follows: 50 mM Tris (MB6025, Meilunbio), 10 mM CaCl2 (MB2581, Meilunbio), 150 mM NaCl (MB2471-1, Meilunbio), and 0.05% Brij 35 (MB4841-1, Meilunbio). The cleavage of IFX, UST, and VDZ was evaluated by immunoblotting using a rabbit anti-human IgG Fc Antibody (Thermo Fisher Scientific).

### Histopathology.

Histopathological analysis by H&E staining was performed as we previously described (39).

### HA Immunohistochemistry.

Sections from patients with IBD were deparaffinized with xylene, followed by dehydration with ethanol. Sections were then treated with 3% H_2_O_2_ at room temperature for 20 minutes in the dark. Antigen retrieval was performed in 10 mM sodium citrate (pH 6.0). The sections were blocked with 5% FBS at 37°C for 30 minutes and then were incubated with biotinylated HA binding protein (385911, Sigma-Aldrich, 1:500) at 4°C overnight in a humidity box. On the next day, HA binding was detected using the ABC-AP-Kit (AK-5200, Vector Laboratories), followed by counterstaining with hematoxylin for 15 seconds. Thereafter, the sections were dehydrated with ethanol, were made transparent with xylene, and then photographed under a light microscope.

### Measurement of IFX concentration.

Serum IFX concentrations in patients with IBD and colitic mice were measured using the IDKmonitor Infliximab Drug Level ELISA Kit (K9655, Immundiagnostik AG) following the manufacturer’s protocol.

### Primary human macrophages.

Human peripheral blood mononuclear cells from healthy donors were isolated using Ficoll-Hypaque reagent (P9011, Solarbio) by gradient centrifugation. The isolated peripheral blood mononuclear cells were cultured with RPMI 1640 medium containing 10% FBS, 100 U/mL penicillin and 100 μg/mL streptomycin (15140122, Thermo Fisher Scientific), 25 mM HEPES buffer (51558, Sigma-Aldrich), and 50 ng/mL M-CSF (300-25, PeproTech) in a 6-well plate. On day 3 and day 6, fresh medium was added. On day 9, adherent macrophages were trypsinized and reseeded in a 12-well plate. Culture supernatant was collected 24 hours after reseeding and filtered through a 0.4 μm pore size filter. For LPS treatment, macrophages were stimulated with 1 μg/mL LPS (Sigma-Aldrich) for 24 hours, and then culture supernatant was collected and filtered.

### Macrophage depletion.

Mice were i.p. injected with 200 μL clodronate liposomes (F70101C-N, FormuMax Scientific) 2 days before DSS treatment. The injection was performed again 4 days after DSS challenge.

### ELISA.

The levels of inflammatory cytokines were evaluated using ELISA kits for IL-6 (Peprotech), IL-1β (Thermo Fisher Scientific), IFN-γ (Thermo Fisher Scientific), and CXCL10 (R&D Systems) following the manufacturer’s protocols. HA contents were evaluated using the HA ELISA Kit from Cusabio.

### Immunofluorescence.

The deparaffinization, dehydration, antigen retrieval, and blocking steps were performed as mentioned above. Slides were incubated with primary antibodies at 4°C overnight in a humidity box. The following antibodies were used: anti-MMP3 (sc-21732, Santa Cruz Biotechnology), anti-HAS2 (sc-514737, Santa Cruz Biotechnology), anti-CD68 (I10341A, Biolynx), or anti-αSMA (80008-1-rr, Proteintech). On the next day, the slides were incubated with secondary antibodies (HKI0029, HaokeBio) for 50 minutes at room temperature. Afterward, the signal was amplified with the Flare570 Signal-Amplification Kit (HKI0015, HaokeBio) following the manufacturer’s protocol. The slides were counterstained with DAPI, washed, and then mounted using an antifading solution. Sections were imaged using a Nikon Eclipse C1.

### Statistics.

All data are presented as mean ± SEM. Unpaired 2-tailed Student’s *t* test, 1-way ANOVA followed by Kruskal-Wallis test and Dunn’ correction, or Spearman’s rank correlation test was performed where appropriate. *P* < 0.05 was considered statistically significant.

### Study approval.

Experiments using human specimens were performed under the approval of the Medical Ethics Committee of Sir Run Run Shaw Hospital, Zhejiang University School of Medicine. Informed consent was obtained from all participants. Animal studies were performed according to protocols approved by the Animal Ethics Committee of Sir Run Run Shaw Hospital, Zhejiang University School of Medicine.

### Data availability.

All data generated in this study are included in the article and supplemental materials. The raw data are available from the corresponding authors upon reasonable request. The GEO data (GSE16879) can be obtained from the official website of GEO database (https://www.ncbi.nlm.nih.gov/geo/query/acc.cgi?acc=GSE16879). Values for all data points in graphs are reported in the Supporting Data Values file.

## Author contributions

Study concept and design was provided by PX and QC. Experiments and data analysis were performed by XC, ZC, WX, HW, Y Zhao, YH, KG, YL, HC, Y Zhang, RL, CM, YF, and PX. Clinical samples were collected and processed by QC, ZC, XC, Y Zhao, RL, HC, HW, and ZS. The manuscript was written by ZC, XC, WX, PX, and QC. Financial or technical support was provided by PX, QC, RL, ZS, YF, and HC. Technical support was provided by XL. Validation was provided by ZC, XC, WX, PX, and QC. The order of the co–first authors was decided by the temporal order in which the authors started performing this study.

## Supplementary Material

Supplemental data

Unedited blot and gel images

Supporting data values

## Version 1 01/09/2025

Electronic publication
