# Supplementary material for: Targeting hyaluronan synthesis enhances the therapeutic effectiveness of biologics in inflammatory bowel disease
Source: JCI Insight. 2025 Jan 9;10(1):e180425. doi: 10.1172/jci.insight.180425 (PMC11721290; doi:10.1172/jci.insight.180425)
Supplement: Supplemental data [file jciinsight-10-180425-s184.pdf]

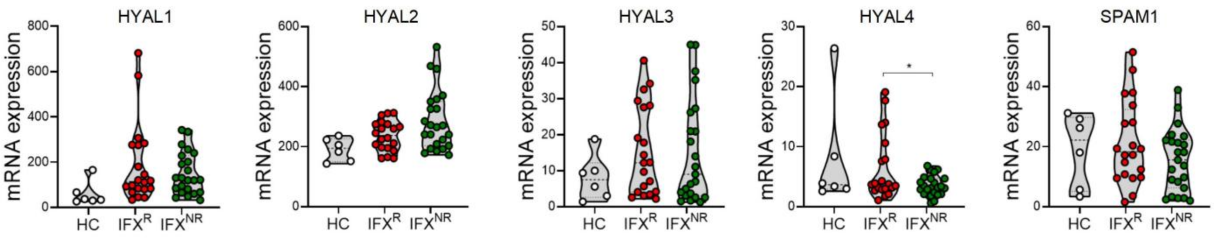

Supplemental Figure 1. The expression of genes encoding HA degradation enzymes in the intestinal mucosa of IFX^R and IFX^{NR} IBD patients was analyzed using GSE16879 dataset. *$p < 0.05$, unpaired, two-tailed Student's t test.

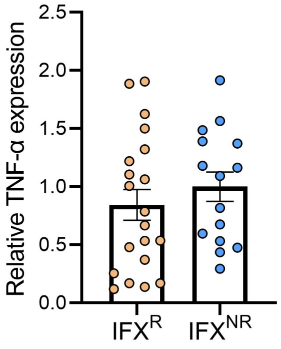

Supplemental Figure 2. The expression of mucosal TNF- α in IFX^R and IFX^{NR} IBD patients was analyzed using our in-house cohort.

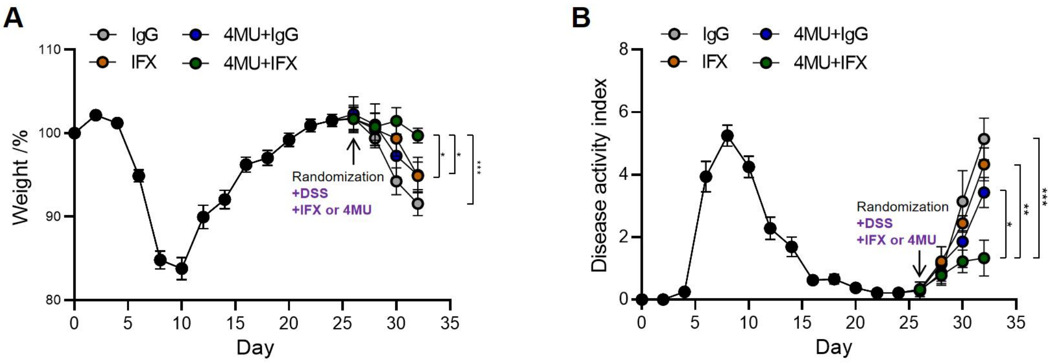

Supplemental Figure 3. The impact of 4MU on IFX effectiveness was evaluated in a chronic colitis model. Mice were fed 2.5% DSS to establish the chronic colitis model. Starting from the day 0 of the second round, mice were administered with IFX, 4MU, or in combination. Body weight changes (A) and disease activity index (B) were monitored. *p < 0.05; **p < 0.01; ***p < 0.001, ANOVA followed by Kruskal-Wallis test and Dunn' correction.

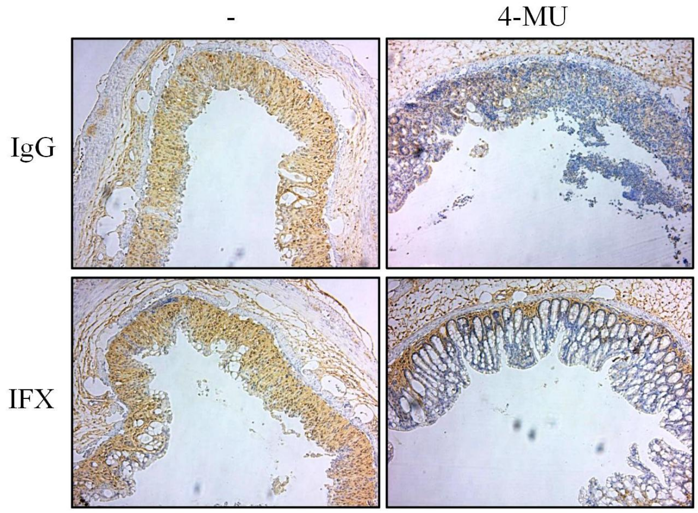

Supplemental Figure 4. Mice given 2.5% DSS were treated with IFX, 4MU, or in combination, HA contents in the colons of colitic mice were evaluated by immunohistochemistry.

Supplemental Table 1

Patient characteristics for IFX cohort. SD = Standard deviation.

	IFX^{R} (n=20)	IFX^{NR} (n=15)
Subtype (CD/UC)	14/6	7/8
Sex (Female/Male)	14/6	8/7
BMI (mean ± SD)	18.89±2.63	19.81±2.02
Smokers/Non-smokers	0/10	0/10
Family history (Yes/No)	0/10	0/10

Patient characteristics for VDZ cohort.

	VDZ^{R} (n=10)	VDZ^{NR} (n=10)
Subtype (CD/UC)	8/2	8/2
Sex (Female/Male)	7/3	5/5
BMI (mean ± SD)	21.22±2.32	20.68±2.99
Smokers/Non-smokers	0/10	0/10
Family history (Yes/No)	0/10	0/10

Supplemental Table 2

Primer Sequences (5’-3’)

human β-Actin F	CATGTACGTTGCTATCCAGGC
human β-Actin R	CTCCTTAATGTCACGCACGAT
human MMP3 F	CTACTTGGGGAGATCGGATGT
human MMP3 R	CTGGGCCACTTTAAGTCTAGC
human CD68 F	GGAAATGCCACGGTTCATCCA
human CD68 R	TGGGGTTCAGTACAGAGATGC
human MMP12 F	GGAATCCTAGCCCATGCTTTT
human MMP12 R	CATTACGGCCTTTGGATCACT
human HAS1 F	TCAAGGCGCTCGGAGATTC
human HAS1 R	CTACCCAGTATCGCAGGCT
human HAS2 F	CTCTTTTGGACTGTATGGTGCC
human HAS2 R	AGGGTAGGTTAGCCTTTTCACA
human HAS3 F	CAGCCTATGTGACGGGCTAC
human HAS3 R	CCTCCTGGTATGCGGCAAT
mouse β-Actin F	GGCTGTATTCCCCTCCATCG
mouse β-Actin R	CCAGTTGGTAACAATGCCATGT
mouse HAS2 F	CATCTGTGGAGATGGTGAAGGTC
mouse HAS2 R	AGCCATCCAGTATCTCACGCTG
mouse MMP3 F	ACATGGAGACTTTGTCCCTTTTG
mouse MMP3 R	TTGGCTGAGTGGTAGAGTCCC
