# Supplementary figures and images for: Targeting hyaluronan synthesis enhances the therapeutic effectiveness of biologics in inflammatory bowel disease
Source: JCI Insight. 2025 Jan 9;10(1):e180425. doi: 10.1172/jci.insight.180425 (PMC11721290; doi:10.1172/jci.insight.180425)

Figure 2D

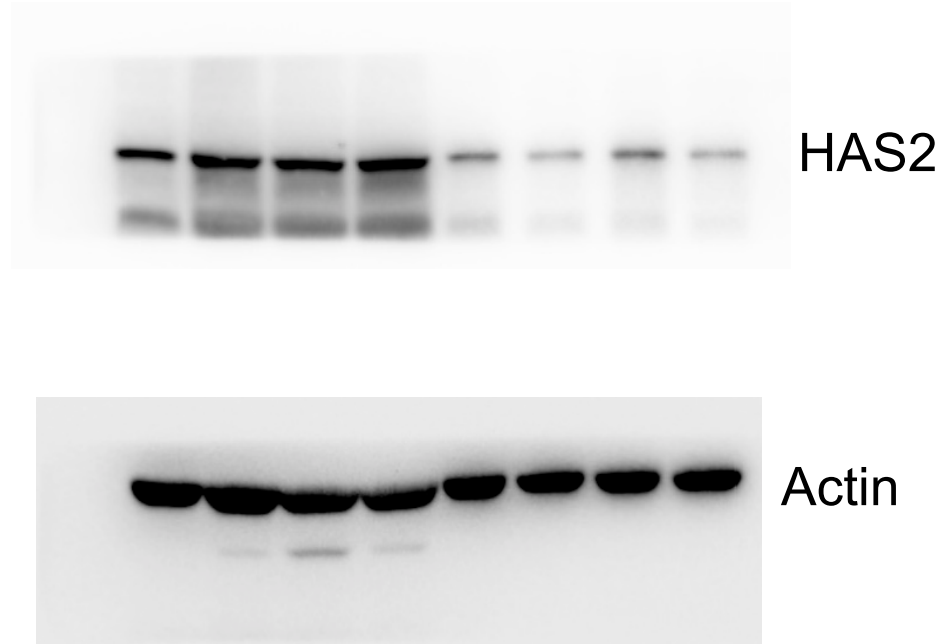

Figure 4F

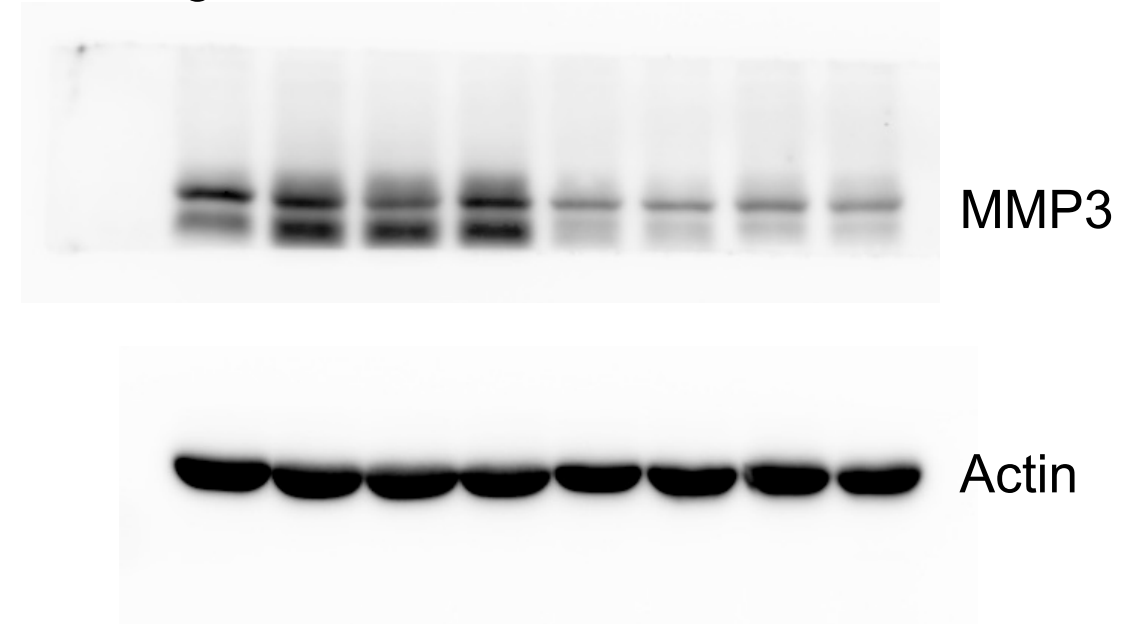

Figure 4H

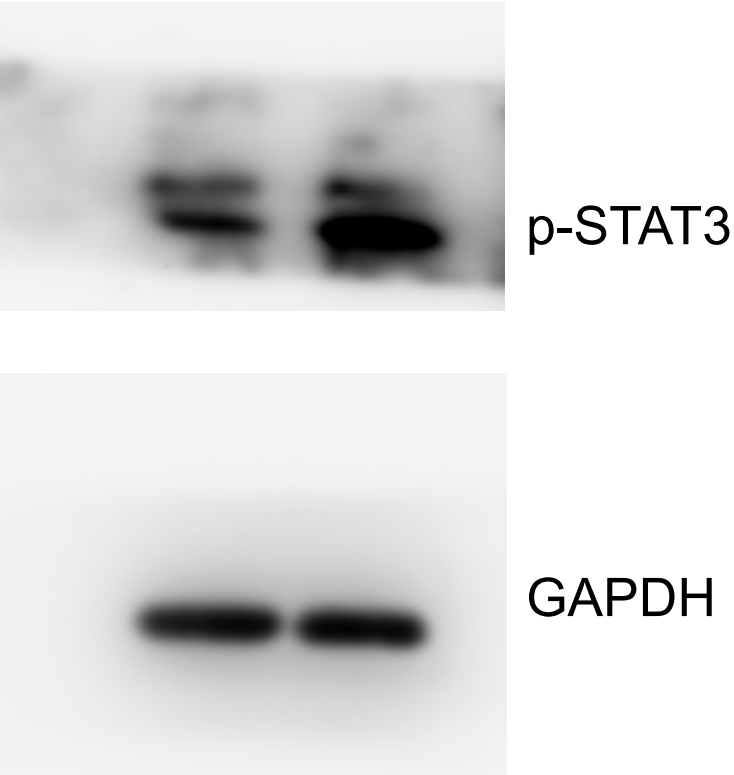

Figure 5E

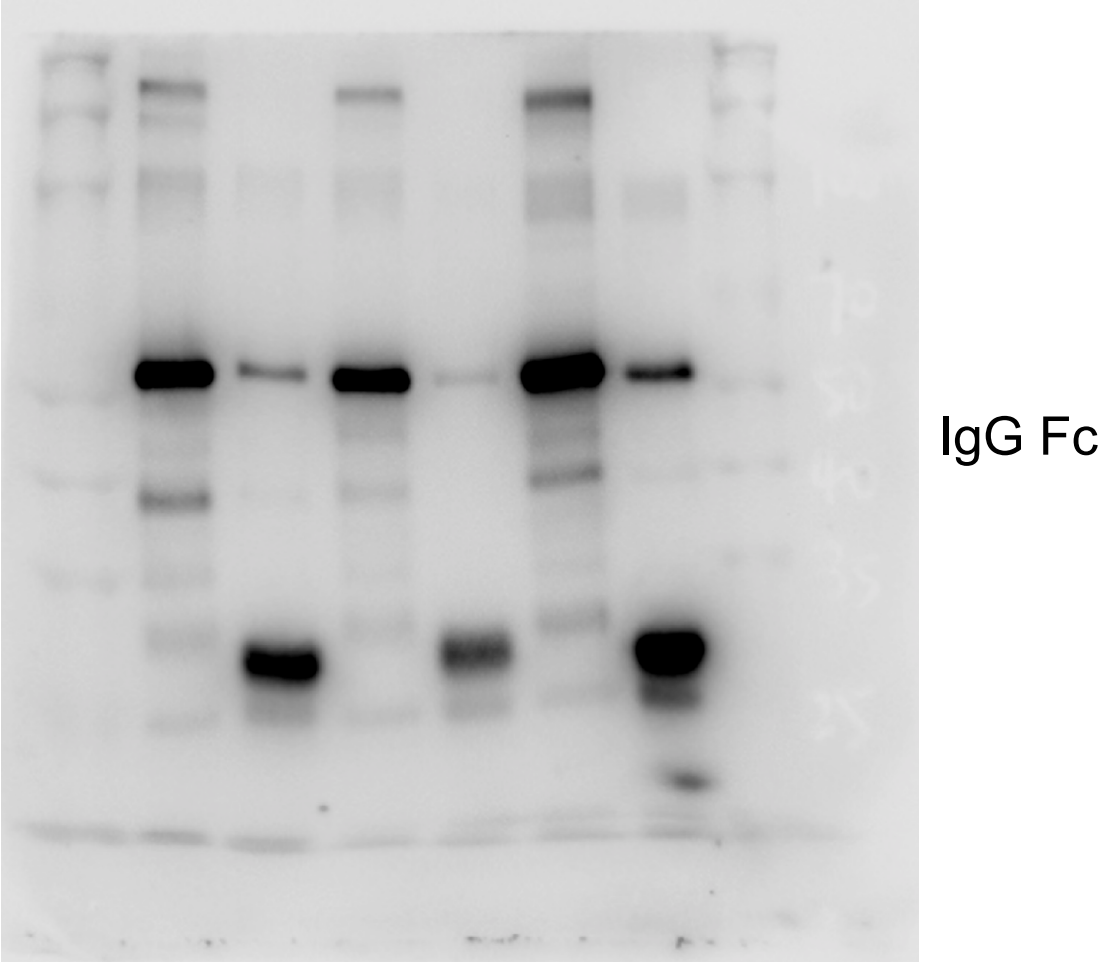

Supplement: Unedited blot and gel images [file jciinsight-10-180425-s185.pdf]
